# A rare triad of morning glory disc anomaly, moyamoya vasculopathy, and transsphenoidal cephalocele: pathophysiological considerations and surgical management

**DOI:** 10.1007/s10072-021-05221-2

**Published:** 2021-04-06

**Authors:** Marco Pavanello, Pietro Fiaschi, Andrea Accogli, Mariasavina Severino, Domenico Tortora, Gianluca Piatelli, Valeria Capra

**Affiliations:** 1 Department of Neurosurgery, IRCCS Istituto Giannina Gaslini Children’s Hospital, Genoa, Italy; 2 Section of Neurosurgery, IRCCS Ospedale Policlinico San Martino, Genoa, Italy; 3 Dipartimento di Neuroscienze, Riabilitazione, Oftalmologia, Genetica e Scienze materno infantili (DINOGMI), IRCCS Ospedale Policlinico San Martino, Università di Genova, Genoa, Italy; 4 Neuroradiology Unit IRCCS Istituto Giannina Gaslini Children’s hospital, Genoa, Italy

**Keywords:** Morning glory, Anomaly, Basal encephalocele, Moyamoya, Phenotypic spectrum, OFD1, Revascularization

## Abstract

Morning glory disc anomaly is a congenital abnormality of the optic disc and peripapillary retina reported as an isolated condition or associated with various anomalies, including basal encephaloceles and moyamoya vasculopathy. However, the co-occurrence of these three entities is extremely rare and the pathogenesis is still poorly understood. Moreover, data on the surgical management and long-term follow-up of the intracranial anomalies are scarce. Here, we describe the case of a 11-year-old boy with morning glory disc anomaly, transsphenoidal cephalocele, and moyamoya vasculopathy, who underwent bilateral indirect revascularization with encephalo-duro-myo-arterio-pericranio-synangiosis at the age of 2 years, and endoscopic repair of the transsphenoidal cephalocele at the age of 6 years. A rare missense variant (c.1081T>C,p.Tyr361His) was found in *OFD1*, a gene responsible for a X-linked ciliopathy, the oral-facial-digital syndrome type 1 (OFD1; OMIM 311200). This case expands the complex phenotype of OFD1 syndrome and suggests a possible involvement of *OFD1* gene and *Shh* pathway in the pathogenesis of these anomalies.

## Introduction

Morning glory disc anomaly (MGDA) is a congenital abnormality of the optic nerve characterized by an enlarged and funnel-shaped excavation of the peripapillary fundus with glial tissue overlying the center of the disc, an annulus of chorioretinal pigment, and spoke-like vessels radiating outward from the edge of the anomalous disk [1]. Most cases are isolated and unilateral, affecting females twice than males. A constellation of associated anomalies has been described, including midline craniofacial defects, cephaloceles, agenesis of the corpus callosum, renal malformations, and intracranial vascular anomalies [2, 3]. In particular, subjects with MGDA may present an increased prevalence of intracranial fetal variants, arterial agenesis, or moyamoya disease [2, 4]. Moyamoya vasculopathy is characterized by steno-occlusive changes at the terminal portion of the internal carotid arteries with formation of an abnormal vascular network at the base of the brain [5–9]. Recently, Brodsky et al. suggested that there may be a common denominator between MGDA and moyamoya vasculopathy, since the absence of central retinal vasculature in MGDA gives rise to compensatory collateralization of chorioretinal anastomoses acting as a “moyamoya” bypass system within the distal optic nerve [10]. Of note, moyamoya syndrome is different from primary or idiopathic moyamoya disease as it develops secondary to an underlying disorder such as Down syndrome, sickle cell disease, PHACE syndrome, neurofibromatosis or other RASopathies, or may occur after brain radiation therapy [5, 11]. Accordingly, the surgical management of Moyamoya syndrome might be complex, since the long-term clinical course may be complicated by the frequently unpredictable natural history of the associated disorder [7, 11, 12].

Another interesting association of MGDA is with basal cephaloceles, rare malformations characterized by evagination of intracranial structures through a congenital defect of the anterior skull base and dura [13]. In particular, MGDA are associated with sphenoidal cephaloceles and milder midline cranial defects related to the persistence of the craniopharyngeal canal [3, 4, 14, 15]. The craniopharyngeal canal is a well-corticated defect through the midline of the sphenoid bone from the sellar floor to the anterosuperior nasopharyngeal roof, likely originating from incomplete closure of the Rathke pouch, the precursor of the adenohypophysis [16]. Recently, the persisting embryonal infundibular recess, a funnel-shaped liquoral space of the third ventricle floor extending into the pituitary stalk, has also been reported in association with MGDA [17]. Intriguingly, this malformation might be considered the mildest form of Rathke pouch’s defective closure.

Of note, the co-occurrence of MGDA, moyamoya vasculopathy, and basal cephalocele is extremely rare with only three patients described so far [18–20]. The pathogenesis of these combined anomalies is poorly understood, and the genetic cause is still unknown. Moreover, data on the surgical management and long-term follow-up of moyamoya vasculopathy and basal cephalocele in MGDA are scarce. Notably, information on the optimal timing and long-term effects of surgical revascularization in these subjects might have important counseling implications for parents.

Here, we describe the clinical, neuroimaging, and genetic features of an 11-year-old boy with MGDA, mild midfacial defects and digital anomalies, who underwent bilateral indirect revascularization for moyamoya vasculopathy at the age of 2 years and transsphenoidal repair of a spheno-nasopharyngeal cephalocele at the age of 6 years.

## Clinical report

**Fig. 1:**
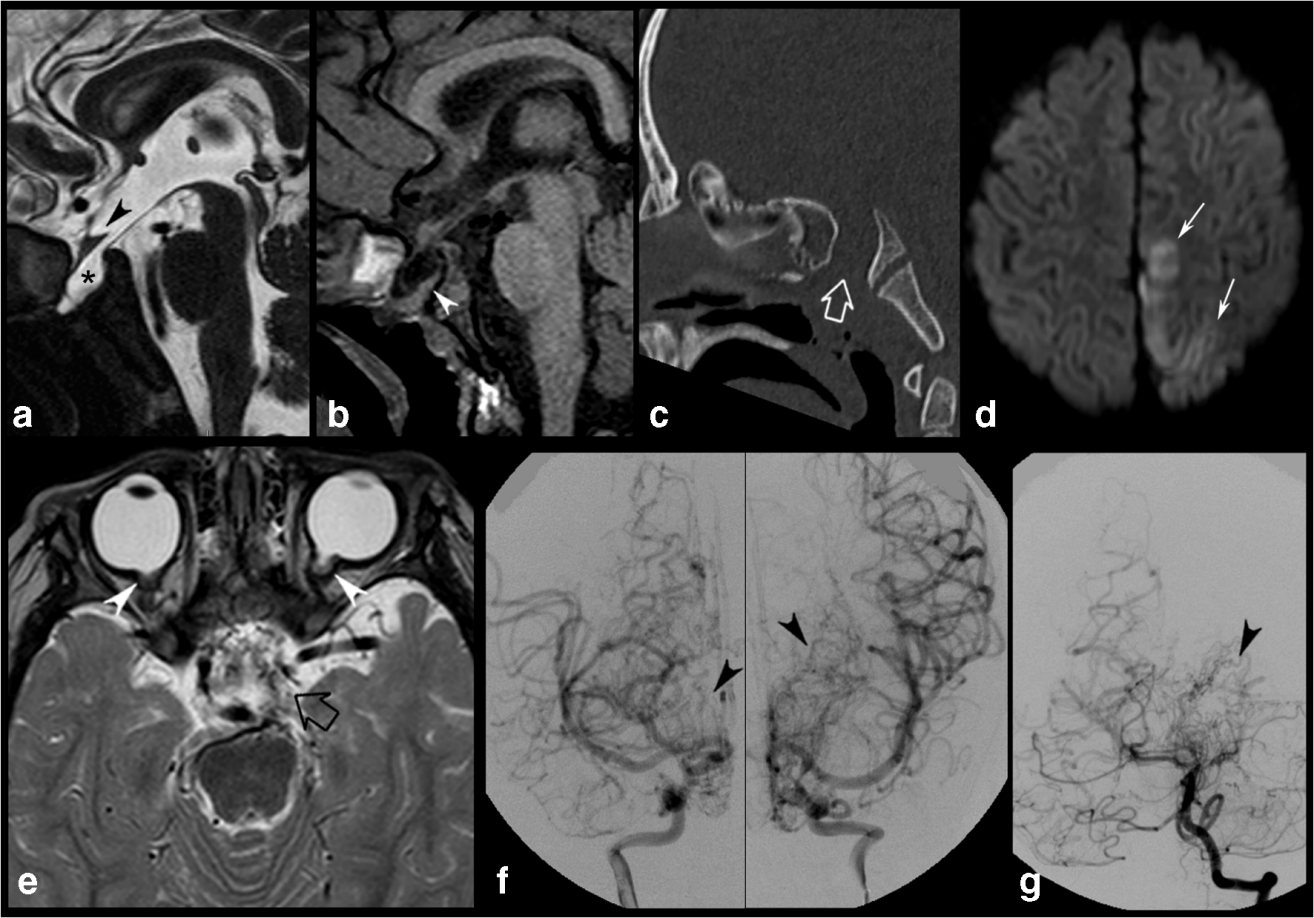
Preoperative imaging findings of the patient. **a**, **b** Brain MRI and **c** CT scan performed at 6 months of age. **d**, **e** Brain MRI. **f**, **g** Digital subtraction Aagiography performed at 2 years of age. Sagittal T2 heavily weighted (DRIVE) (**a**) and T1-weighted (**b**) images reveal a spheno-nasopharyngeal cephalocele with herniation of the pituitary gland and third ventricle through a sphenoid bone defect. The pituitary gland is flattened against the clivus (arrowhead, **b**). The optic chiasm is distorted and displaced downward (arrowhead, **a**). **c** 3D CT scan, sagittal MPR reconstruction demonstrates a large craniopharyngeal canal (empty arrow). **d** Axial diffusion-weighted imaging performed at 2 years of age demonstrates an acute ischemic infarct in the left medial parietal and frontal lobes (thin arrows). **e** Axial T2-weighted image shows multiple tiny flow voids in the basal cisterns consistent with moyamoya vascular collaterals (empty arrow). Note the bilateral optic nerve funnel-shaped excavations with associated abnormal soft tissue and discontinuity of the normal uveoscleral coat at the optic nerve insertions, in keeping with MGDA (arrowheads). Preoperative digital subtraction angiography, frontal views, internal carotid artery injections (**f**) and left vertebral artery injection (**g**) confirm the diagnosis of moyamoya disease with bilateral stenosis of the supraclinoid carotid arteries, anterior cerebral arteries, and left posterior cerebral artery, and the typical “puff of smoke” sign due to the presence of compensating vascular networks (arrowheads)

The boy is the first child to a healthy Brazilian mother and an Italian father. He was born full-term without perinatal complications. During the neonatal period, rotational nystagmus was noted in both eyes. Fundus examination revealed bilateral MGDA. Brain MRI and head CT performed at 6 months of age showed bilateral MGDA associated with a small transsphenoidal cephalocele (Fig. 1a–c). At that time, no cerebral vascular anomalies were found, except for the presence of right persistent trigeminal artery and fetal origin of the left posterior cerebral artery. The abdominal ultrasound was normal. At the age of 2 years, he presented with acute drowsiness, transient right hemiparesis, gait disturbances, and hyperreflexia. Brain MRI with arterial MR angiography (MRA) depicted an acute ischemic infarct in the left posterior medial frontal and parietal lobes and bilateral moyamoya vasculopathy with involvement of both anterior and posterior circulation (Fig. 1d, e). Continuous heparin infusion (15UI/kg/h) was immediately started and then switched to antiplatelet therapy (aspirin dose 4 mg/kg die). A digital subtraction angiography confirmed the diagnosis of a Suzuki stage IV moyamoya vasculopathy (Fig. 1f, g).

**Fig. 2:**
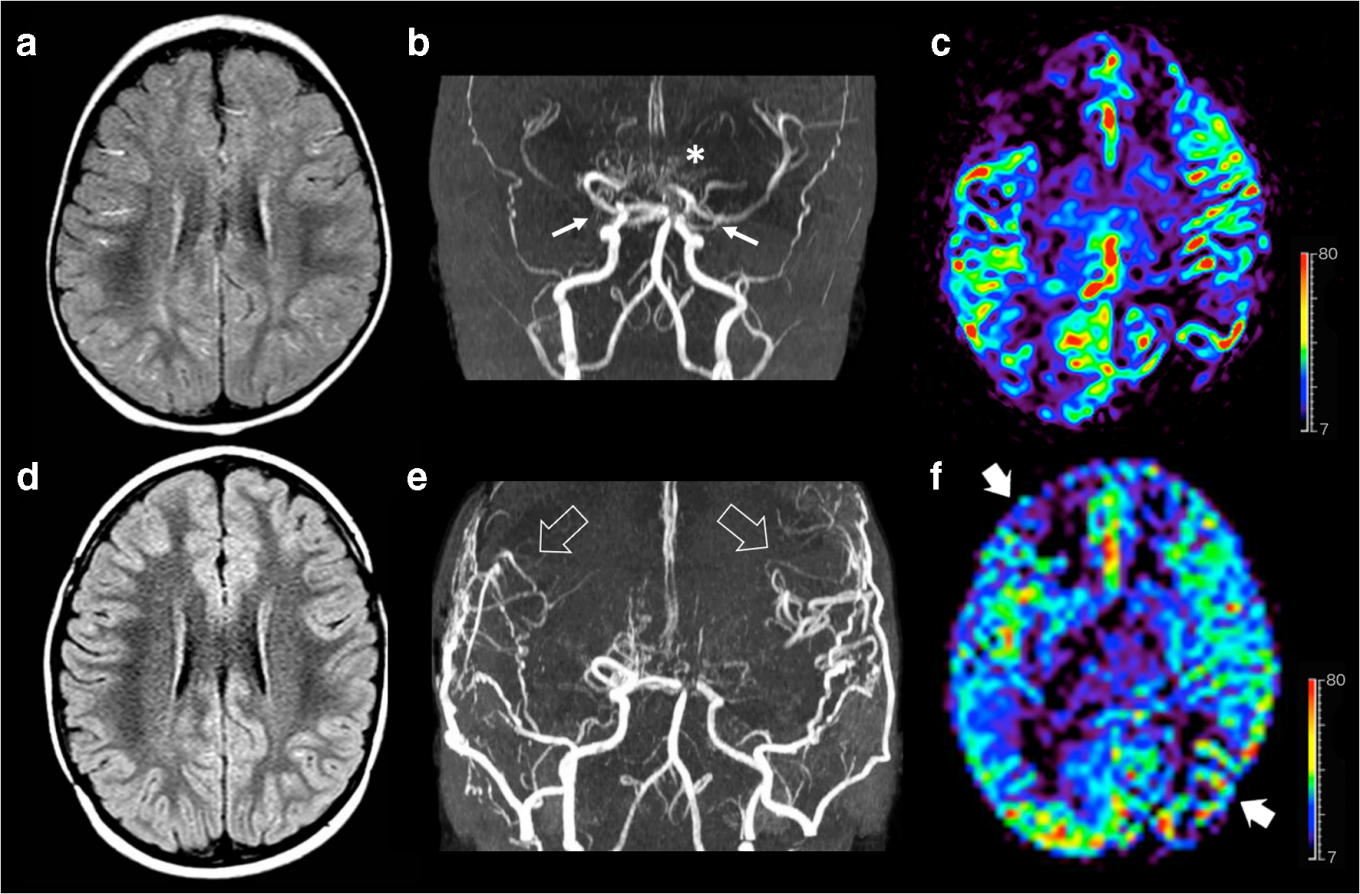
Brain MRI studies with MRA and arterial spin labeling (ASL) perfusion acquired before (**a**–**c**) and 1 year after surgery (**d**–**f**). **a** Axial FLAIR image reveals linear high signal intensity along the cortical sulci in both cerebral hemispheres, in keeping with diffuse prominent leptomeningeal collaterals. **b** Arterial MRA shows bilateral moyamoya vasculopathy with stenosis of distal portion of both internal carotid arteries (arrows), severe stenosis of right middle cerebral artery, posterior circulation involvement, and basal moyamoya collateralization (asterisk). **c** ASL perfusion map reveals hypoperfusion of both middle cerebral artery territories, in particular of the right frontal lobe and left parietal lobe. **d** Axial FLAIR image after surgery demonstrates resolution of the leptomeningeal collaterals. **e** Postoperative MRA demonstrate development of pial collateralization in the sites of surgical revascularization (arrowheads). **f** ASL perfusion map unravels marked improvement of the CBF in the middle cerebral artery territories after surgery (thick arrows). Color bar indicates ml/min/100 g for ASL-CBF.

Three weeks after the ischemic event, the patient underwent left indirect revascularization with encephalo-duro-myo-arterio-pericranio-synangiosis (EDAMPS), followed 3 months later by right EDAMPS. The patient showed a good recovery and regression of neurological symptoms. Serial follow-up brain MRI and MRA with perfusion studies showed bilateral development of pial collateralization in the sites of surgical revascularization with marked improvement of brain perfusion (Fig. 2).

At the age of 6 years, the patient started complaining of headache with no other neurological symptoms or endocrine deficits. Brain MRI and MRA did not show new ischemic lesions, vascular changes, or worsening of the brain perfusion, while the transsphenoidal cephalocele was increased in size. Therefore, transsphenoidal repair of the spheno-nasopharyngeal cephalocele was performed. In details, using endoscopic endonasal approach, the sac was isolated and partially removed, and the defect was reconstructed with a nasoseptal flap to prevent the risk of cerebrospinal fluid leaks. The postoperative course was uneventful, and the headache resolved. The postoperative brain MRI demonstrated marked reduction of the cephalocele volume.

Two years after surgery, the patient showed reduced growth velocity with low IGF1 levels (30 ng/ml, n.v. 61–356). Glucagon stimulation test showed a GH peak value of 0.38 ng/ml, compatible with GH deficiency. Treatment with recombinant human growth hormone (rhGH) was therefore started. At the age of 9 years, TSH level was found to be low and treatment with levothyroxine was started.

**Fig. 3:**
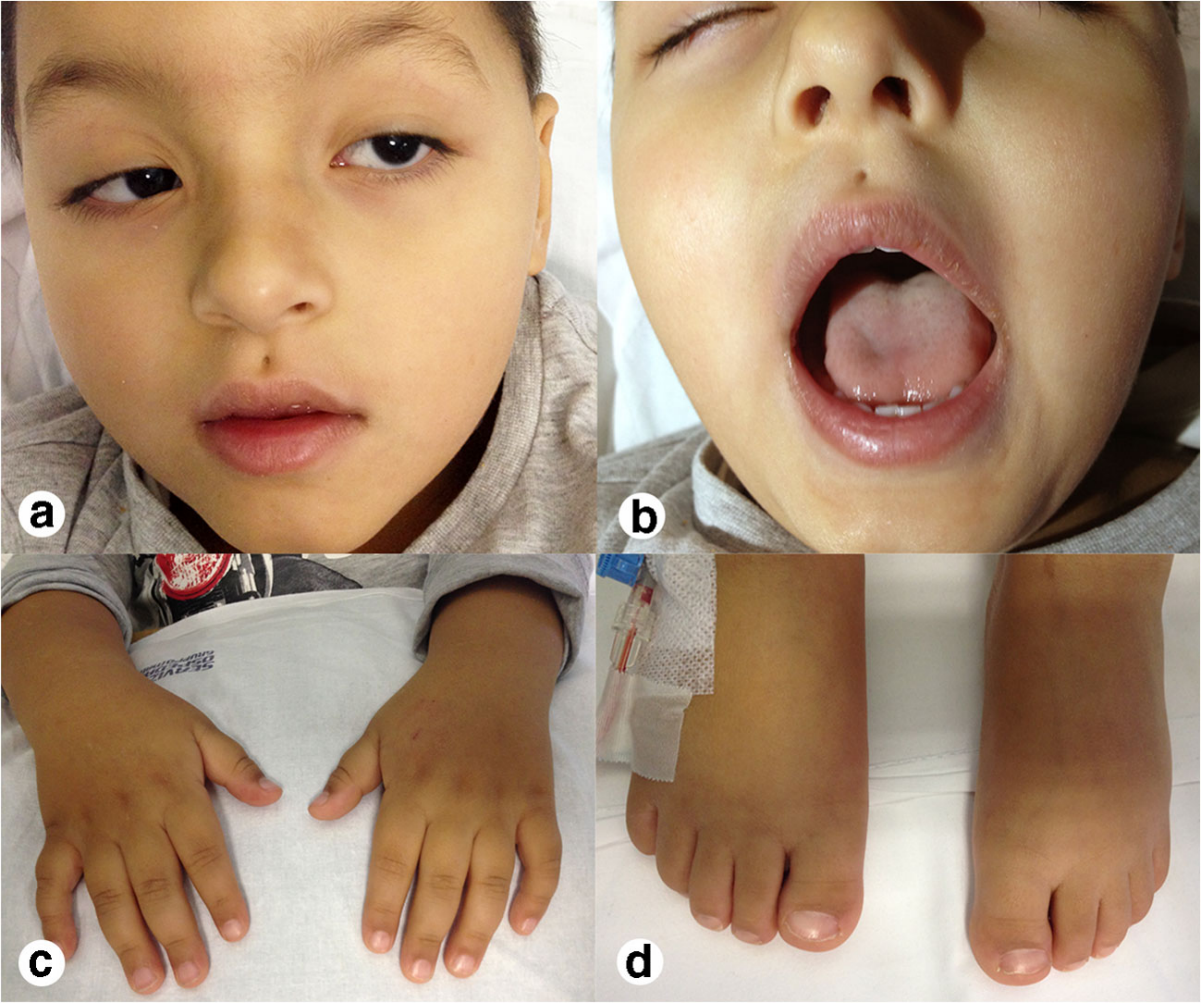
Clinical findings of the patient. **a**, **b** Ptosis, hypertelorism, a pit in the midline of the philtral groove. **c** Bilateral brachydactyly and clinodactyly of the V digit

At last evaluation, at the age of 11 years, his height was 98 cm (< 3rd centile), his weight was 18.5 kg (75th centile), and he had an OFC of 48.5 cm (< 3rd centile). On physical examination, he presented rotational nystagmus, ptosis, hypertelorism, a pit in the midline of the philtral groove, short neck, brachydactyly, clinodactyly of the V digit bilaterally, short toes, broad hallux bilaterally, and micropenis (Fig. 3). Brain MRI and MRA demonstrated stable vascular features and brain perfusion. Psychomotor evaluation revealed a normal IQ (104) according to Wechsler Preschool and Primary Scale of Intelligence (WPPSI). Array-CGH detected a de novo 1.39 Mb duplication on 11q14.1 band of chromosome 11 (77,888,679–79,278,347x3) (hG19) that was considered not pathogenic. Given the midline defects of skull base and philtrum associated with minor anomalies of hands and feet, reminiscent of the oral-facial-digital syndrome type 1 (OFD1; OMIM 311200), target sequencing of *OFD1* was carried out. A novel hemizygous missense variant, c.1081T>C,p.Tyr361His (NM_003611.2), was detected in the boy and in his healthy mother. This variant has never been reported in the literature or in any public database, including gnomAD. The p.Tyr361His is a change of an evolutionary conserved amino acid and is predicted deleterious by several predictive tools (PolyPhen, SIFT, FATHMM, CADD, DANN, GERP, LRT, Provean, MetaLR, MetaSVM, MutationAssessor). Although c.1081T>C,p.Tyr361His is classified as a variant of uncertain significance according to the American College of Medical Genetics and Genomics (ACMG) guidelines, it holds two criteria, namely PM2 (absent in gnomAD) and PP3 (multiple lines of computational evidence supporting a deleterious effect) that suggest a possible pathogenic role. However, given the lack of other clinical features of OFD1 and the finding of MGDA and moyamoya vasculopathy never reported before in this syndrome, we performed whole-exome sequencing (WES) through a trio-based approach to seek other possible pathogenic variants. Data were processed using in-house analytical pipelines that include publicly available tools and custom scripts. We looked at non-synonymous exonic and splicing variants with a minor allele frequency ≤ 0.001 in gnomAD database and we did not identify any possible deleterious variants in any other genes.

## Discussion

The etiology of MGDA, basal cephaloceles, and moyamoya vasculopathy is poorly understood, also due to the lack of genetic testing in subjects with this peculiar phenotype. Here, we describe the case of an 11-year-old boy with MGDA, mild midfacial defects, digital anomalies, moyamoya vasculopathy, and sphenoid cephalocele, who harbored a new rare missense variant in *OFD1*. This gene is responsible for the oral-facial-digital syndrome type 1 (OFD1; OMIM 311200), a X-linked-dominant male-lethal ciliopathy characterized by malformation of face, oral cavity and digits, cystic kidney disease, and a wide spectrum of brain malformations [21]. Of note, *OFD1* mutations have recently been associated to novel phenotypes outlining the wide phenotypic spectrum of ciliopathies [22]. However, sphenoidal encephalocele, MGDA, and moyamoya vasculopathy have never been associated with *OFD1* mutations. To date, the majority of *OFD1* mutations are small insertions or deletions, whereas missense mutations are rarely reported [22]. Therefore, we hypothesize that the missense variant in our patient might have contributed to the milder clinical features, as the function of the resulting protein might not be critically damaged.

As for other ciliary proteins, it has been demonstrated in vivo and in vitro that *Ofd1* inactivation leads to defective sonic hedgehog (Shh) [23]. Interestingly, the phenotypic analysis of *Ofd1-/-* mouse demonstrates a crucial role of Shh signaling in the closure of the craniopharyngeal canal [24]. In mammals, the craniopharyngeal canal (also known as buccohypophyseal canal) usually closes in the early stages of development. Remarkably, *Ofd1-/-* mutant mouse show a wide basisphenoid defect with inferiorly displaced pituitary tissue [24]. Moreover, a small sphenoid ossification defect below the pituitary gland and an incomplete median cleft of the upper lip similar to the present case have been observed in a patient carrying an *OFD1* mutation [24]. Taken together, these data suggest that missense *OFD1* variants may lead to midline cranial defects, such as the persistence of the craniopharyngeal canal and transsphenoidal cephaloceles. Of note, hedgehog signaling also regulates optic fissure and stalk formation during vertebrate eye morphogenesis [25]. *Ofd1* inactivation by injection of antisense morpholinos in zebrafish causes the incomplete fusion of the choroid fissure of the eye resulting in a retinal coloboma similar to MGDA [26]. Interestingly, retinal atrophy and thin optic nerves have been detected in a small cohort of females with *OFD1* variants [27, 28]. Therefore, we speculate that *OFD1* might also indirectly interfere in the closure of the posterior aspect of the fetal optic fissure, leading to MGDA. Finally, disruption of the Shh signaling may also be implicated in moyamoya vasculopathy, taking into account the pivotal role of this gene in vessel formation and remodeling. Indeed, an increased vascularization of neuroectoderm after Shh overexpression has been reported [29]. On the other hand, the attenuation of Shh response by blocking Shh signal causes hemorrhage and damage in branching and remodeling of the aortic arches [30]. Interestingly, moyamoya vasculopathy has been described in other ciliopathies, such as autosomal dominant polycystic kidney disease and Kartagener syndrome [31, 32], indicating that primary cilia have important roles in cardiovascular diseases [33].

Regarding the clinical and neuroimaging features of this patient, it is noteworthy that the first brain MRI performed at the age of 6 months did not reveal any vascular abnormality, except for two fetal variants. Subsequently, the rapid onset and progression of moyamoya vasculopathy lead to a cerebral ischemic infarct at the age of 2 years of age. This raises an important point related to the screening and surveillance of intracranial vascular anomalies in individuals with MGDA and basal cephaloceles. Indeed, moyamoya arteriopathy is associated with the highest odds of having recurrent ischemic strokes in children, with relevant consequences for the clinical outcome and quality of life [34]. Moreover, early-onset moyamoya vasculopathy demonstrates rapid disease progression and results in poorer clinical outcomes [35]. Therefore, as suggested by Seung-Ki Kim [35], early surgery seems indicated for young patients with moyamoya vasculopathy, although controlled studies with long-term follow-up monitoring are needed to define the actual benefits of revascularization in these cases. In the present case, we decided to perform bilateral revascularization surgery considered both the ischemic complication and the extended bilateral reduction of cerebral blood flow identified at brain MRI perfusion studies [36]. Due to the inadequate caliber of the superficial temporal artery, instead of a combined direct-indirect revascularization, we opted for EDAMPS, which was bilaterally performed with a 3-month interval. Indeed, it has been demonstrated that indirect revascularization improves cerebral hemodynamics and reduces the incidence of subsequent ischemic events in children with moyamoya vasculopathy [8, 37, 38]. Accordingly, in the present case, we noted regression of the ivy sign on FLAIR images and marked improvement of cerebral blood flow on ASL studies after surgery. Of note, these results were stable 9 years after direct revascularization, and the patient did not experience additional cerebral ischemic infarcts.

Headaches may arise from intra­ and/or extracranial pain­sensitive structures such as skin, muscles, blood vessels, dura mater, and cranial nerves. In the present case, the onset of headache at 6 years of age was not associated with worsening of the moyamoya vasculopathy and/or brain perfusion. Conversely, an increase in size of the transsphenoidal cephalocele was observed, in the absence of endocrinological problems. Of note, headache has been reported in subjects with transsphenoidal cephaloceles, likely related to the mass effect or the traction on adjacent structures, such as the optic chiasm and pituitary gland [39]. Moreover, in a retrospective study, Fleseriu et al. demonstrated improvement of intractable headache after surgery of small sellar lesions, such as pituitary microadenomas or Rathke cleft cysts [28]. Therefore, we decided to repair the spheno-nasopharyngeal cephalocele using a transsphenoidal approach. Indeed, despite the continuous advancement of technical surgeries, transsphenoidal approach is still considered the gold standard for skull base surgery [40–43]. According to Yang et al., the transsphenoidal approach may be the optimal treatment for the management of many symptoms related to transsphenoidal cephaloceles, and patients with milder preoperative symptoms have a better prognosis [43]. In the present patient, the headache resolved after transsphenoidal surgery. On the other hand, pituitary hormone deficiency emerged 2 years after the intervention. We cannot exclude that hypopituitarism might be related to the surgical intervention. However, the long interval from surgery and the onset of similar symptoms in non-operated subjects indicate that hormonal problems are part of the natural history of sphenoidal cephaloceles, as previously reported [44].

## Conclusion

In conclusion, this case provides insights on the potential role of *OFD1* in MGDA, basal cephalocele, and moyamoya vasculopathy. However, functional studies are needed to shed light on the role of this gene and Shh signaling in the pathogenesis of this rare triad. Regarding the clinical and surgical management, this case underlines the importance of screening and surveillance of moyamoya vasculopathy in individuals with MGDA and basal cephaloceles, especially considered the possibility of early revascularization that might improve the clinical outcome. Finally, surgical management of the basal cephalocele might be pondered in the presence of symptoms that may not be attributed to other causes.

## Abbreviations

MMD: Moyamoya disease
MGDA: Morning glory disc anomaly
OFD: Oral-facial-digital
WPPSI: Wechsler Preschool and Primary Scale of Intelligence
BHC: Buccohypophyseal canal
GH: Growth hormone
TSS: Transsphenoidal surgery
MRI: Magnetic resonance imaging
TSH: Thyroid-stimulating hormone
FLAIR: Fluid-attenuated inversion recovery
EDAMPS: Encephalo-duro-myo-arterio-pericranio-synangiosis
MRA: Magnetic resonance angiography
